# The Long Outer-Hair-Cell *RC* Time Constant: A Feature, Not a Bug, of the Mammalian Cochlea

**DOI:** 10.1007/s10162-022-00884-w

**Published:** 2023-02-01

**Authors:** Alessandro Altoè, Christopher A. Shera

**Affiliations:** 1 Caruso Department of Otolaryngology, University of Southern California, Los Angeles, CA USA; 2 Department of Physics & Astronomy, University of Southern California, Los Angeles, CA USA

**Keywords:** Outer hair cell, *RC* time constant, Amplification, Noise, Distortion

## Abstract

The cochlea of the mammalian inner ear includes an active, hydromechanical amplifier thought to arise via the piezoelectric action of the outer hair cells (OHCs). A classic problem of cochlear biophysics is that the *RC* (resistance-capacitance) time constant of the hair-cell membrane appears inconveniently long, producing an effective cut-off frequency much lower than that of most audible sounds. The long *RC* time constant implies that the OHC receptor potential—and hence its electromotile response—decreases by roughly two orders of magnitude over the frequency range of mammalian hearing, casting doubt on the hypothesized role of cycle-by-cycle OHC-based amplification in mammalian hearing. Here, we review published data and basic physics to show that the “*RC* problem” has been magnified by viewing it through the wrong lens. Our analysis finds no appreciable mismatch between the expected magnitude of high-frequency electromotility and the sound-evoked displacements of the organ of Corti. Rather than precluding significant OHC-based boosts to auditory sensitivity, the long *RC* time constant appears beneficial for hearing, reducing the effects of internal noise and distortion while increasing the fidelity of cochlear amplification.

## Introduction

To boost the sensitivity and dynamic range of hearing, the mammalian inner ear embeds a physiologically vulnerable active process that amplifies sound-evoked motions as they propagate as waves along the cochlear spiral [1, 2]. Although operation of the cellular amplifiers requires prestin-based somatic outer-hair-cell (OHC) electromotility [3–6], serious doubts persist about whether the piezoelectric mechanism can respond with the speed and vigor necessary to boost the power carried by waves of high frequency. This vexing subject has been the focus of much research and debate, starting shortly after the discovery of OHC electromotility [7, 8] and continuing to the present day [9–11].

There are two distinct and largely independent issues. The first, of more recent vintage, concerns the kinetics of prestin, which may not be fast enough to support high-frequency electromotility [10]. The second, more widely appreciated due to its long shadow, arises because OHC electromotility is driven by transmembrane voltage [12]. Electrically, the OHC membrane consists primarily of a resistance (*R*) and a shunt capacitance (*C*) that combine to create a low-pass filter with time constant $\tau =RC$. With one exception [13], both direct [7, 14] and indirect [11, 15] observations suggest that in the basal, high-frequency region of the cochlea, the time constant $\tau$ is much larger than the characteristic oscillation period of the sound-evoked mechanical response of the basilar membrane (BM). Equivalently, the cutoff frequency of the OHC low-pass filter is much lower than the maximum frequency of hearing. Consequently, the low-pass filtering is presumed to render the oscillatory component of the OHC receptor potential too small to subserve high-frequency amplification. Dubbed the outer-hair-cell “*RC* time-constant problem” [16]—or, more compactly, the “*RC* problem” [11]—the dilemma has prompted the search for compensatory mechanisms [17–20] while challenging the biophysical relevance of “cycle-by-cycle” electromotility to cochlear amplification [8, 9].

Discussions of the *RC* problem—and, more generally, of the relevance of high-frequency OHC motility to cochlear function—typically revolve around two conflicting positions, positions whose proponents might be dubbed the “*RC* pessimists” and the “*RC* optimists.” On the one hand, the *RC* optimists argue either that the effective *RC* time constant operating in vivo is actually shorter than classically measured (e.g., because the classic measurements were performed in unphysiological conditions; cf. [13]) or that various intervening electromechanical mechanisms act to mitigate or circumvent the *RC* filtering (e.g., [17–23]). On the other hand, the *RC* pessimists maintain that the crippling effects of membrane low-pass filtering are evident not only in a dish but in OHC electrical and mechanical responses measured in vivo, so that the optimists’ proposed solutions to the *RC* problem (and to OHC speed problems more generally) appear, at best, ineffective (e.g., [9, 11, 24]).

In an attempt to place the *RC* controversy in perspective, we analyze recent data on the subject from a third and, we hope, enlightening vantage point. Rather than recounting and reviewing the various proposed mechanisms conjectured to facilitate high-frequency OHC electromotility in vivo, we focus on a much simpler question: As a practical matter, do published in vitro data actually call the significance of OHC high-frequency motility into question? Arguments that infer debilitating high-frequency limits on OHC operation are based on measurements that reveal low-pass filtering in OHC responses (e.g., [10, 11]). But these measurements do not address the functionally more relevant question of whether, despite possible low-pass filtering, high-frequency OHC responses nevertheless remain large enough to enable electromotilty to play a significant role in cochlear mechanics.

Although definitive evaluation of OHC performance requires controlling for the in situ mechanical load that so greatly influences OHC electromechanical behavior (see e.g., [22, 23, 25–27]), in vivo vibrational data from the mouse apex suggest that the OHCs can indeed produce significant active motions at high frequencies (>20 kHz), notwithstanding the fact that their cycle-by-cycle elongation appears low-pass filtered relative to the motion of the BM [15]. In other words, the ratio between OHC and BM motion decreases with frequency, but remains greater than 1 near CF. Furthermore, recent recordings from the gerbil cochlea using high-resolution optical coherence tomography (OCT) suggest that OHC electromotiliy makes a significant contribution to measured cochlear amplification up to at least 50 kHz [28]. Our analysis confirms and extends these suggestions by analyzing a simple OHC model with parameters chosen to represent a “worst-case” scenario. In the section “Pessimistic Model of the *RC* Problem”, we adopt the premises and strictly empirical perspective of the *RC* pessimists. While reviewing the various simplifications involved, we deduce from the literature a “pessimistic” model of high-frequency (50 kHz) OHC operation. In “The Long Time Constant Is Not a Bug”, we compare the output of the pessimistic model with published intracochlear motions [29] and show that, despite attenuation by worst-case *RC* filtering, predicted OHC cycle-by-cycle elongations remain significant relative to the measured motion of the BM and surrounding tissue. By demonstrating that even the worst-case scenario retains ample headroom to subserve high-frequency cochlear amplification, our analysis amounts to a reductio ad absurdum of the pessimists’ case against OHC electromotility.

Possible functional benefits of OHC membrane filtering have occasionally been identified, such as rotating the phase of the OHC response to facilitate the pumping of energy into the traveling wave (e.g., [30])—a curious design from an engineering perspective, since adequate phase rotations can easily be achieved by other means without the need to simultaneously attenuate the voltage drive to the OHC motor [12]. But in the context of the ongoing debate, the long OHC time constant appears almost exclusively as a “bug” or physical limitation of the OHC. Building on the success of our analysis—the solution to the problem of the *RC* time constant is seen in the vanishing of the problem—we switch lenses and look more closely at the advantages that membrane filtering might bring to the hearing organ. These benefits include significant reductions in intracochlear distortion and noise that are the inevitable byproducts of active, nonlinear OHC-based amplification.

Why has the issue of the *RC* time constant dominated discussions of OHC electromotility for more than three decades, even though calculations that might be done in the margins of a *JARO* paper render the problem almost moot? In “Supply and Demand”, we suggest that a narrow focus on supply (i.e., the effects of low-pass filtering on OHC cycle-by-cycle elongation) without considering demand (e.g., the known tonotopic variations of BM displacement near CF) has inflated a secondary problem to primary significance. Our conclusions here complement those recently drawn by Rabbitt [31], who has demonstrated that the same in vitro experiments that reveal low-pass filtered OHC cycle-by-cycle elongations and contractions also indicate that the active OHC power (i.e., the power that the OHC can deliver to the outside world) peaks at high frequencies, well above the apparent limits deduced from the frequency response of OHC cycle-by-cycle elongation.

Finally, just as one swallow does not a summer make, the presence of a low-pass filter, or even an entire flight of them, need not imply low-pass functional operation. We elucidate this analogy and other caveats surrounding *RC*-related problems through the analysis of hypothesized non-amplifying roles for the OHCs and real-life examples.

## Pessimistic Model of the *RC* Problem

### Linearized Model of the OHC Membrane

**Fig. 1:**
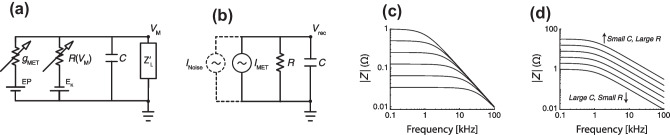
**a** Simplified model of the OHC membrane potential including MET and voltage-dependent basolateral K^+^ channels, the membrane shunt capacitance, *C*, and a phenomenological impedance quantifying the effect of the mechanical load ($Z'_\mathrm {L}$) on the membrane potential (see Appendix 2). **b** Over-linearized model of the OHC receptor potential often used to illustrate the *RC* problem. Whereas the ground in panel **a** represents the perilymph potential, it represents the OHC resting potential in panel **b**. **c**,** d** Magnitude of the electrical impedance (*Z*, Eq. (1)) of the *RC* circuit in panel **b**. Panel **c** plots |*Z*| vs frequency for various values of the time constant ($\tau =RC$) when the capacitance (*C*) is kept fixed; in this case, $\tau$ varies linearly with *R*. Conversely, panel **d** plots |*Z*| when *R* and *C* are covaried with their product, $\tau$, held constant. Together, the two panels demonstrate graphically that at frequencies above the low-pass cut-off frequency, |*Z*| depends entirely on the capacitance and not on the time constant (Eqs. (1 and 2)).

To illustrate the ostensible problems that arise from the electrical properties of the OHC, we outline a simple treatment of the generic electrical model shown in Fig. 1a. In particular, we show that by oversimplifying the physics one can obtain the linearized, low-pass filter model typically employed to question the functional significance of cycle-by-cycle OHC electromotility. Although the resulting model is well known, our derivation here is pedagogical and reviews the various simplifications and assumptions that give rise to the *RC* problem.

Briefly, the mechanically gated mechanoelectrical transduction (MET) current drives the electrical response of the OHC basolateral membrane. The electrical impedance of the basolateral membrane is determined primarily by (i) outward K$^+$ currents, whose collective action is represented by a voltage-dependent resistor [13]; (ii) the membrane capacitance; and (iii) a complex impedance, represented by $Z'_\mathrm {L}$ in Fig. 1a, that captures the electrical effects of the OHC mechanical load [22]. The inclusion of the impedance $Z'_\mathrm {L}$, which is technically a describing function (since the OHC nonlinear capacitance renders the load nonlinear), makes the model of Fig. 1a consistent with published electromechanical models. For example, the load impedance depends not only on the mechanical properties of the load, but also on the OHC electromechanical transducer characteristic. This and other caveats related to the effect of the load and OHC transduction characteristics are reviewed in Appendix 2.

To highlight the problem of the OHC time constant in its most severe form, we now (over-)linearize the model of the OHC membrane. Thus, we ignore the possibly salutary effects of the voltage-dependent basolateral currents [7, 32] and the mechanical load [22]. With these (over-)simplifications, the OHC membrane appears as the parallel combination of a resistance and capacitance, driven by a (MET) current generator (Fig.  1b). (To quantify the effects of various forms of noise on the OHC output, we sometimes find it convenient to introduce a second current generator, in parallel with the MET current.) We provide details of the simplification in Appendix 1.

The electrical impedance of the OHC membrane, defined as the ratio of the oscillating (AC) component of the receptor potential $\tilde{V}_\mathrm {rec}$ and the resulting MET current $\tilde{I}_\mathrm {MET}$, then takes the form of an *RC* (low-pass) filter,

1 $$\begin{aligned} Z(f)=\frac{\tilde{V}_\mathrm {rec}(f)}{\tilde{I}_\mathrm {MET}(f)}=\frac{R}{1+i f/f_{\mathrm {c}}}\;, \end{aligned}$$

where *R* is the resting basolateral resistance and $f_{\mathrm {c}}$ is the 3-dB cut-off frequency ($f_{\mathrm {c}}=[2\pi RC]^{-1}$). Figure 1c, d shows how |*Z*(*f*)| depends on *R* and *C* by varying *R* and $f_{\mathrm {c}}$ (with *C* constant) and by varying *R* and *C* (with $f_{\mathrm {c}}$ constant).

In high-frequency regions of the cochlea, OHCs must operate well above the cut-off imposed by their *RC* time constant. In this limit, where the current flowing through the resistance *R* becomes small compared to that flowing onto the capacitor *C*, the AC receptor potential becomes

2 $$\begin{aligned} \tilde{V}_\mathrm {rec}(f)\approx \tilde{I}_\mathrm {MET}/(i 2\pi f C)\;. \end{aligned}$$

In other words, the “*RC* problem” purported to limit the high-frequency operation of the OHC reduces to the “*C* problem” (©van der Heijden and Vavakou [9]): that is, to the problem of charging the capacitor to a meaningful potential using the available MET current.

Finally, we can relate receptor potential amplitude with OHC cycle-by-cycle elongation ($\Delta _{\mathrm {OHC}}$) via the relation

3 $$\begin{aligned} \Delta _{\mathrm {OHC}}(f)=\tilde{V}_\mathrm {rec}(f)T_\mathrm {EMT}(f)\;, \end{aligned}$$

where $T_\mathrm {EMT}$ represents the frequency-dependent “electro-mechanical” transfer function, defined as the cycle-by-cycle elongation produced per unit AC receptor potential. Although $T_\mathrm {EMT}$ is ideally measured in vivo, we here employ in vitro estimates (see below).

We want to stress that the oversimplified OHC model outlined above serves only to illustrate the *RC* problem and is clearly inadequate to address fundamental questions regarding the biophysics of electromotility (as briefly reviewed in Appendix 2). For example, the model does not account for the physically necessary mutual interaction between the receptor potential and the mechanical load (indeed, the *RC* model cannot technically produce motility at any frequency because it does not deliver mechanical power anywhere). On the other hand, the oversimplified model, by representing OHC cycle-by-cycle motility as a cascade of filters (Eqs. (1–3)), captures the *RC* problem in its most severe form. The model also allows one to “plug-in” measured parameters to assess the severity of purported limitations on high-frequency electromotility. Furthermore, both intracellular electrical recordings [14] and mechanical measurements of OHC motion in vivo appear well explained by a nonlinear transduction function subject to first-order low-pass filtering [11, 15]. Whether (or why) the simple model provides a satisfying phenomenological approximation of OHC function remains controversial for technical reasons. Whereas intracellular recordings can be compromised by the alteration of OHC properties following electrode impalement, published OCT recordings have yet to achieve the spatial resolution needed to accurately estimate the elongation of individual OHCs (but see [28]).

### Worst-Case Parameter Selection

To explore whether the membrane capacitance limits high-frequency operation of the OHC, we apply Eq. (3) to estimate OHC receptor potentials and electromotility in the region of the mouse cochlear tuned to $\sim$50 kHz [29]. (Together with the gerbil base [28], this is the highest frequency region for which systematic mechanical recordings of BM and organ-of-Corti motions are available.) To side-step the many unknowns concerning OHC force production, we compare estimated limits on OHC length changes with in vivo BM displacements. The argument goes as follows: If the low-pass filtered electrical drive to the OHCs can produce motile responses comparable to or larger than the measured motion of the BM, then the OHC response is evidently “large enough,” and membrane filtering does not preclude functionally relevant operation.

When choosing parameter values, we suppress any optimistic bias by purposefully imagining a worst-case scenario. Consider, for example, the well-known tonotopic variation of OHC properties. Whereas OHC lengths and capacitance increase systematically along the cochlear spiral, MET-channel conductances decrease with position [13, 33, 34]. For a given AC receptor potential, long OHCs elongate more than short ones [35]. On the other hand, short cells have smaller capacitance, and are therefore more easily charged. The MET conductance determines the amplitude of the MET current, and hence the magnitude of the OHC electromotile response. Since our goal is to play devil’s advocate by making unfavorable parameter choices, we not only neglect these known tonotopic variations, but purposely counteract them to inflate the apparent severity of the *RC* problem.

We therefore imagine that our hypothetical murine OHCs, operating at the 50-kHz place, are simultaneously (i) short, and therefore limited in their ability to elongate, and (ii) endowed with membrane capacitance and MET currents representative of OHCs from more apical locations with a much lower CF [13]. We thus assume that the available AC MET current ($\tilde{I}_\mathrm {MET}$) is only on the order of 1.5 nA and that the OHC capacitance (*C*) is $\sim$4 pF, values representative of data from the 10-kHz place in rat [13]. Although some studies (e.g., [22, 36]) argue that the effective capacitance of the OHC in vivo is significantly reduced by piezoelectric interaction with the mechanical load, others suggest that the nonlinear dynamics of electric charge moving along the membrane significantly increases the effective OHC capacitance near the potential that maximizes their mechanical response [37]. Here, we take our capacitance value from a 10-kHz OHC, which has roughly double the membrane area [38], and hence double the linear capacitance [39], of an actual 50-kHz cell. Thus, the model is pessimistic enough to accommodate a potential doubling of the effective OHC capacitance in vivo. If the goal were to leverage tonotopic variations to facilitate high-frequency electromotility, our parameter choices reflect a genuinely counterproductive approach.

Estimating OHC motion requires knowing the in situ relation between AC receptor potentials and OHC elongation. Unfortunately, both the absence of direct in vivo measurements of electromotile responses from basal OHCs and the complex dependence of in vitro OHC electromotility on experimental parameters [10, 16, 40] render this relationship uncertain. Frank et al. [35] found that isolated (unloaded) OHCs driven by a sinusoidal voltage expand and contract without substantial attenuation up to frequencies significantly higher than their CF, although these results have recently been questioned [10]. Nevertheless, one obtains that the electromotile response of an unloaded OHC is on the order of 0.5 nm/mV at 50 kHz, either by extrapolating to a $\sim$15 $\mu$m basal mouse OHC from the data [35] or by taking the (extrapolated) data [10] at face value (see Fig. 10B of [10]). Indeed, Rabbitt [31] recently demonstrated that with respect to high-frequency OHC function, the differences between the two studies are negligible.

In addition, in situ recordings from the excised temporal bone of the guinea pig [41] show that electrical excitation of basal OHCs (CF$\,\sim \,$24 kHz) produces motion of the surrounding tissue (i.e., the lower surface of the TM and RL) on the order of $\sim$2 nm/mV at frequencies up to CF, rolling off gently to about 1 nm/mV around 50 kHz. (Near CF their recordings show moderate resonances and anti-resonances.) Interestingly, OHC-induced responses measured in situ [41] are larger than those reported in vitro: the data of [35] indicate that a $\sim$30 $\mu$m long OHC (i.e., the length of basal OHCs reported by [41]) produces responses on the order of 1.2 nm/mV, only 60% of the RL response measured in situ (2 nm/mV). (These differences may be due to in situ interactions with the mechanical load and/or to the maintenance of a more physiological resting potential than occurs in the microchamber; both can influence the magnitude of the electromotile response [10].) More recently, Levic et al. [42] used measurements of OHC nonlinear capacitance [43] to estimate that the basal mouse OHC response is about 1.7 nm/mV at 50 kHz. Taking all the evidence together, a reasonably conservative estimate would have basal OHCs driven at 50 kHz capable of elongating on the order of 1 nm/mV, whether acting in isolation or aided by the mechanics of the surrounding tissue (i.e., ${T_\mathrm {EMT}(50\,\mathrm{kHz})=1\,\mathrm{nm/mV}}$ in Eq. (3)).

## The Long Time Constant Is Not a Bug

### Worst-case OHC Produces Sufficient Motion

Combining the conservative parameter values discussed above, we find (Eq. (2)) that our worst-case OHC is capable of elongating on the order of 1.25 nm at 48 kHz ($\Delta _{\mathrm {OHC}}\sim 1\,\mathrm {nm/mV}\cdot 1.5\,\mathrm {nA}/(2\pi \cdot 48\,\mathrm {kHz}\cdot 4\,\mathrm {pF})$). For comparison, vibrational data from the 48-kHz region of the mouse cochlea [29] indicate that in vivo mechanical movements are close to this value at sound levels of 60–70 dB SPL (at 48 kHz, BM and RL responses at 70 dB SPL are less than 3 nm). In these experiments, both BM and RL gain at CF decrease with level above 30 dB SPL (where BM motion is $\sim$0.2 nm) and are dramatically reduced above about 70 dB. Thus, despite low-pass filtering, our approximate upper bound on the magnitude of OHC electromotile cycle-by-cycle elongation (1.25 nm) is much larger than the BM displacements measured at low sound levels and is comparable to BM displacement at moderately high levels. Note that if this comparison had come out differently—if our approximate upper bound on OHC electromotility had been much less than the displacement of the BM and surrounding structures—then assertions that cycle-by-cycle somatic motility appears too weak to influence intracochlear motions at high frequencies would be intuitively compelling. As it turns out, however—and notwithstanding a number of unfavorable assumptions—the comparison demonstrates that the low-pass filtering of the OHC membrane does not, in practice, impose limitations on the cell’s high-frequency response that compromises its ability to make significant contributions to measured cochlear amplification.

### AC Receptor Potentials Are Small Near the Threshold of Hearing

Unfortunately, it is not easy to determine, on theoretical grounds, how much the OHCs need to elongate and contract in order to provide the wave amplification required at any given sound level. However, for a complementary perspective, we can run the analysis outlined above “in reverse” by using Eq. (2) to infer the voltage and current necessary to produce OHC motile responses comparable in magnitude to the BM vibrations observed near the threshold of hearing. Linear extrapolation of the low-level reference data [29] indicates that a 48-kHz tone at 10-dB SPL—a sound level somewhat smaller than the threshold of the most sensitive auditory-nerve fibers [44]—evokes BM responses of $\sim$20 pm, requiring voltage swings on the order of 20 $\mu$V, and corresponding MET currents of roughly 25 pA. This current is on the order of the AC current mediated by one MET channel. (A single MET channel in the mid-turn of the rat cochlea mediates a current of $\sim$12 pA and the channel conductance increases with CF [33]; assuming $\sim$100 MET channels per OHC [33], then a single channel in the worst-case OHC model mediates a current of $\sim$3 nA/100 $=$ 30 pA.) Although these estimates may appear small, they are consistent with OHC AC receptor potentials (10–30 $\mu$V) recorded in vivo in the 16-kHz region of the guinea pig at sound levels near the neural threshold measured in the same preparation [45]. Because the invasive approach necessary in such experiments reduces cochlear gain [45], extrapolating the AC potentials to even lower sound levels (e.g., to thresholds typical of uncompromised animals, as recently attempted [9]) is problematic. Additionally, the perforation produced by the microelectrode in vivo has a major impact on hair-cell electrodynamics (see e.g., [46]), with the likely result being an underestimate of the true physiological value of the AC potential [47].

### OHC Electrical Noise Is Small Where It Matters

**Fig. 2:**
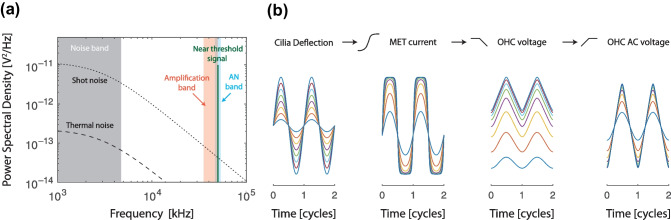
**a** Estimated power spectral density of the inherent electrical noise (thermal and shot) at the OHC output compared to that of a 50-ms, 50-kHz tone pip with an amplitude sufficient to elicit OHC electromotile responses comparable to BM motions at near-threshold sound levels. Power spectral densities were calculated assuming that the OHC membrane cut-off frequency is 3 kHz and the resting MET channel open probability is 30% (see Appendix 2). The three shaded areas indicate (i) the equivalent rectangular bandwidth (ERB) of the OHC output noise (gray; 1.57 times larger than the 3-dB bandwidth of the *RC* filter); (ii) the approximate frequency band of BM amplification at the 50-kHz place (pink; $\sim$ [35,50] kHz [29]); and (iii) the ERB of a 50-kHz mouse auditory-nerve-fiber tuning curve (blue; calculated assuming a quality factor of 8 [44]). Although the overall noise power at the output of a single OHC can be large compared to the signal, this power is confined to low frequencies—far away from relevant ranges of signal detection and amplification—by the low-pass filtering of the OHC. **b** Simple model illustrating how OHC low-pass filtering improves the fidelity of the hearing organ. From left to right: Sinusoidal deflection of the OHC stereocilia produces a distorted MET current via an asymmetric, saturating sigmoidal nonlinearity. The OHC receptor potential is obtained from the MET current by low-pass filtering, in accordance with the simple model (Eq. (1)). The oscillating (AC) component of the OHC voltage is obtained by high-pass filtering the OHC voltage. Whereas the MET current saturates and becomes highly distorted as the stereociliary input increases, the OHC voltage appears much less distorted, and the AC response is nearly sinusoidal. This same model captures the mechanical distortions measured near the OHCs in the organ of Corti [15]

Can such small voltage excursions and the resulting motile responses have functional relevance in the presence of noise? Multiple noise sources contribute to the OHC transmembrane voltage, including various forms of hydromechanical noise that influence the motion of the stereocilia (see e.g., [48, 49]). Although the magnitudes of these noise sources have yet to be fully characterized, their effects can be explored in the model by introducing a current source whose output represents the sum of the various forms of white (flat power spectral density), pink (1/*f*), and brown ($1/f^2$) noise present in the cochlea. As an example, Fig. 2a compares the power spectral density of a 50-kHz, near-threshold signal (as estimated above from the model) with estimates of the inherent OHC electrical noise—thermal noise and “shot noise” caused by the random gating of the MET channels—assuming an *RC* time constant of 50 $\mu$s (cut-off frequency of 3 kHz). See Appendix 3 for details of the calculations. Noting that behavioral hearing thresholds are well predicted by a neural threshold criterion corresponding to a firing-rate increase of 1 spike per 50 ms above spontaneous rate [50, 51], we took the example signal to be a 20-$\mu$V sinusoid of 50-ms duration. The resulting signal power spectral density is more than 2 orders of magnitude above the electrical noise floor. Since the power spectral density of a tone pip equals the tone power times its duration, Fig. 2a demonstrates that even extremely short signals (<0.5 ms) remain above the noise. Interestingly, the existence of multiple rows of OHCs at each longitudinal location—across which the electrical noise is presumably incoherent—effectively decreases the minimum signal duration required to elicit a response above the noise (by a factor of 3, assuming 3 rows of OHCs and that each row contributes equally to amplification).

## The Long Time Constant Is a Feature

We have seen that the OHC shunt capacitance *C* and associated low-pass filtering appear not to preclude significant OHC motion at high frequencies. But might this filtering be of functional benefit to hearing rather than merely not being harmful? When discussions of OHC electromotility are dominated by the question of whether OHCs can operate at high frequencies, the possible benefits of low-pass filtering by the OHC membrane receive scant attention. Among the handful of potential benefits brought about by the OHC shunt capacitance are (i) a strategic rotation of the OHC response phase, so that OHCs can readily pump energy into the traveling wave [30]; and (ii) the possibility that the membrane capacitance might conspire with the inertial component of the mechanical load to convert the membrane low-pass into a resonant band-pass filter [22, 36]. In this section, we discuss additional benefits that low-pass membrane filtering might bring to the hearing organ, focusing on intracochlear noise and distortions. We elucidate these benefits using the simple *RC* model, consistent with recent in vivo experimental data indicating that the OHC transduction machinery manifests the presence of a first-order low-pass filter [11, 15].

Accurate estimates of the effects of membrane filtering on OHC electromechanical responses in vivo certainly require a better understanding of the kinetics of prestin and the frequency-dependent interactions between the mechanical and electrical domains in the fluid-coupled cochlear spiral (see Appendix 2). Nonetheless, we expect that our general remarks about the benefits of including a first-order low-pass filter in the OHC machinery—elucidated here using the simple *RC* model—will apply to the real cochlea under a broad range of conditions. Specifically, we expect our conclusions to remain valid (i) whether or not the observed low-pass filtering in the OHC response is dominated by the membrane time constant or by other complicating factors, such as interactions with the mechanical load; (ii) to the extent that the membrane capacitance does not interact with the mechanical load to create a peaked resonance (as reviewed by [52], there are as yet no signs of OHC resonance in intracochlear recordings); and (iii) at sufficiently high frequencies, regardless of the simplifying assumptions used to derive the model (see Appendix 2).

### The Long Time Constant Reduces Noise Where It Matters Most

The OHC model in Fig. 1b implies that the OHC basolateral membrane filters noise in the same way that it filters the driving MET current (i.e., the “signal”). Consequently, the signal-to-noise ratio (SNR) measured in small frequency bands around the stimulus frequency largely mirrors the ratio of the stimulus-driven MET current to the noise current in the considered band. This is an important but often neglected aspect of OHC transduction: while the total power of the inherent noise can be comparable to or larger than the signal [9], the noise power is distributed over a much larger bandwidth than the frequency range relevant for both cochlear amplification and the detection performed by the inner hair cell and auditory nerve. As a consequence, the overall noise power in the relevant frequency band for high-frequency signal detection and amplification is small (Fig. 2a). Indeed, in the base of the cochlea, the low-pass filtering performed by the OHC membrane largely confines the noise to tail frequencies, where it has been shown (both empirically and theoretically) that OHC motile and electrical responses are largely uncoupled from the traveling wave (e.g., [53–56]).

Additionally, membrane low-pass filtering attenuates the shot-noise power at the OHC output by about 11 dB (see Appendix 4). The reason is simple: stochastic gating of the MET channels happens on time scales much shorter than the *RC* time constant, and hence a significant fraction of the random gating current is attenuated by membrane low-pass filtering. Interestingly, shot noise is a form of quantization noise, and cascading a high-sample-rate, low-resolution digital signal with a low-pass filter is a well-known strategy employed in telecommunication systems to reduce quantization noise and synthesize low-noise, high-resolution analog signals (e.g., 1-bit digital-to-analog converters; see [57]).

### The Long Time Constant Reduces Cochlear Distortion

Regarding the OHC as a filter operating on sinusoidal signals may overlook important aspects of OHC function. It is now a classic result that the MET transduction function is a highly nonlinear and saturating function [14, 47], implying that the MET current approaches a square wave for sufficiently large excursions of the stereocilia. When the *RC* time constant is larger than the stimulus period, the response remains relatively “clean” despite the highly distorted input, as illustrated in Fig. 2b using a stereotyped OHC model derived from recent measurements of intracochlear distortions in the mouse apex [15]. Thus, the OHC low-pass filter increases the fidelity of the hearing organ by reducing harmonic distortions over a wide range of frequencies. For example, at stimulus frequencies above the OHC membrane cut-off, the amplitude of the *N*th harmonic is attenuated by $6(N-1)$ dB relative to the response at the fundamental ($N=1$). Recently, Peterson and Heil [58] proposed an analogous role for the low-pass filtering present in the IHC mechano-to-neural transduction machinery. Just as in our simple OHC model (Fig. 2b), IHC low-pass filtering reduces the distortion introduced by the saturating MET nonlinearity, thereby increasing the fidelity and temporal precision of auditory-nerve responses [58].

## Discussion

### Supply and Demand

**Fig. 3:**
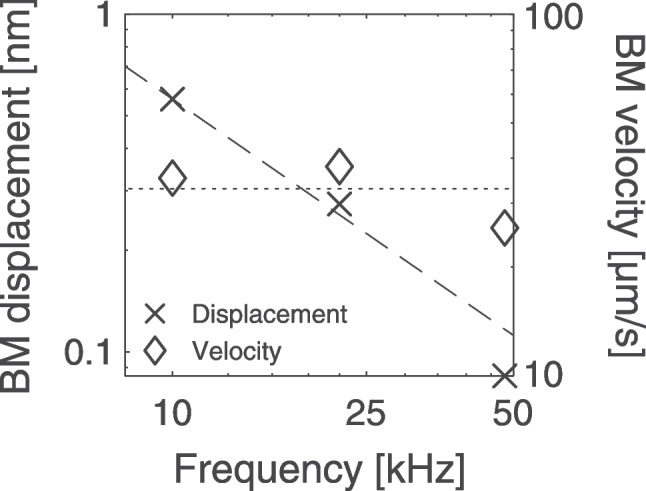
Approximate scaling of BM vibration in the mouse cochlea. The symbols give BM displacement ($\times$) and velocity ($\Diamond$) measured using CF tones of 20 dB SPL (where BM responses are approximately linear) at CFs of 9 kHz [55], 22 kHz (unpublished data courtesy of J. B. Dewey), and 48 kHz [29]. The lines show that the tonotopic variation of near-CF BM displacement and velocity appears nearly inversely proportional to CF (dashed line) and constant (dotted), respectively

Simple arguments suggest that our results, derived from data at $\sim$50 kHz, extend to even higher frequencies. With fixed parameters, the model predicts that the OHC motile response decreases in a manner inversely proportional to frequency. But the same is true both for the expected displacement of the sensory tissue and for the noise at the OHC output (see above). The data in Fig. 3 demonstrate that near-CF BM displacement in the mouse decreases almost linearly with CF. Thus, the functionally relevant metric is not the absolute magnitude of OHC electromotility but the *ratio* of OHC displacement to that of the BM and surrounding tissue. So long as supply and demand remain commensurate, disruptions to the supply-chain alone need not create hardship.

As a corrollary, Fig. 3 implies that BM velocity at the peak of the traveling wave is approximately independent of CF—precisely what modelers have suggested for more than 40 years [59, 60]. Arguments against a significant role for OHC high-frequency motility focus heavily on the low-pass characteristic of OHC displacement brought about by the *RC* time constant and, more recently, by concerns about prestin kinetics [9, 10]. However, assuming that OHC elongation depends linearly on membrane voltage, the membrane acts as a first-order high-pass filter for OHC velocity. In other words, membrane filtering does little to modify the frequency dependence of OHC velocity above $f_{\mathrm {c}}$.

It is worth elaborating on this point using our simplified treatment. Note that the cycle-by-cycle OHC elongation velocity, $\nu$, is proportional to the product of frequency and OHC elongation displacement. The voltage-displacement relation $T_\mathrm {EMT}$ is a function of frequency and cell length, and we therefore write it as the product $T_\mathrm {EMT}(f)=T_\mathrm {EMT_0}\times T_\mathrm {motor}({f})$, where $T_\mathrm {EMT_0}$ represents the low-frequency asymptote for OHC elongation and the function $T_\mathrm {motor}(f)=T_\mathrm {EMT}(f)/T_\mathrm {EMT_0}$ quantifies the filtering due to the kinetics of the prestin motor. Combining Eqs. (2 and 3), we can express peak OHC velocity at $\mathrm {CF}$ (${\nu }_\mathrm {\mathrm {CF}}$) as

4 $$\begin{aligned} {\nu }_\mathrm {\mathrm {CF}}\propto \frac{I_\mathrm {MET}\times T_\mathrm {EMT_0}\times T_\mathrm {motor}({\mathrm {CF}})}{C}\;, \end{aligned}$$

where $\propto$ indicates proportionality. Importantly, the factor of 1/*f* coming from the membrane impedance (Eq. (2)) cancels when considering OHC velocity. Because OHCs at different locations are nearly cylinders with the same radius but varying length [38], the OHC membrane area, and thus the linear capacitance, is expected to vary roughly in proportional to OHC length. Empirical data suggest that $T_\mathrm {EMT_0}$ is also approximately proportional to OHC length (see Fig. 3 of [35]). Therefore,

5 $$\begin{aligned} {\nu }_\mathrm {\mathrm {CF}}\sim {I_\mathrm {MET}(\mathrm {CF}) \times T_\mathrm {motor}({\mathrm {CF}})}\;, \end{aligned}$$

implying that the variation of near-CF OHC velocity along the cochlear spiral is determined primarily by the variation in available MET current and by the amount of “filtering” introduced by the kinetics of the prestin motor. Although $I_\mathrm {MET}$ is known to increase with CF [13, 33, 47], the tonotopic variation of $T_\mathrm {motor}(\mathrm {CF})$ remains to be determined (and understood) in situ. Published estimates from isolated OHCs, however, suggest that it is approximately constant, independent of $\mathrm {CF}$ [35]. While our simplified treatment cannot address the question of whether and how the OHCs amplify signals in vivo—for example, it ignores important factors such as the frequency dependence introduced by the OHC mechanical load—it nonetheless highlights an important expectation deduced from the available data: near-CF OHC velocity (or, equivalently, the ratio of OHC cycle-by-cycle elongation to BM displacement, see Fig. 3) likely increases with $\mathrm {CF}$.

Again, consider the worst-case scenario and assume, naively, that the low-pass voltage-displacement relations observed in isolated apical OHCs [10] apply without modification to basal cells in vivo. Even in this pessimistic case, the combined effects of *RC* filtering and sluggish prestin kinetics would decrease OHC velocity by only about a factor of 2 between 50 kHz—where our analysis reveals that OHC elongation appears sufficient—and 100 kHz (i.e., well beyond the hearing range of common laboratory animals). Counteracting this modest decrease (e.g., via the known increase of $I_\mathrm {MET}$ with CF) hardly seems prohibitive, especially considering the number of unfavorable assumptions necessary to make the problem appear compelling in the first place. An unwarranted focus on supply alone rather than on its relation to demand—on OHC electromotility and its low-pass characteristic rather than on relative OHC and BM vibration magnitudes versus CF—has inflated the apparent severity of the *RC* problem.

### The Scaling-Symmetric OHC

In the real cochlea, OHC properties vary systematically along the tonotopic axis. Nevertheless, many prominent variations in cochlear response features, such as the change in tuning sharpness with CF, can reasonably be accounted for by models in which the action of the OHCs is assumed scaling symmetric and thus—aside from the progressive decrease in CF—essentially identical at all locations [61, 62]. (Some deviations may occur at the extreme apex near the helicotrema [63].) As we noted in “Supply and Demand”, a principal functional consequence of the tonotopic gradient of OHC length—reduced OHC velocity at higher CFs—is effectively counteracted by the gradients of OHC capacitance. How and whether the tonotopic gradient of MET current [13, 47], which act to increase OHC velocity at higher CFs, is equalized by additional mechanisms that might reduce OHC velocity (e.g., the mechanical load and/or the kinetics of the prestin motor) remain open questions. Nonetheless, our analysis indicates that a scaling symmetric model of the OHC action appears physically plausible. Interestingly, Rabbitt and Bidone [64] reach similar conclusions after evaluating more factors (e.g., the kinetics of the MET channels and the mechanical load) than are considered here.

Although the scaling-symmetric OHC appears theoretically compelling, the empirical reality remains hotly debated [27, 40, 65, 66] and the resolution still pending. We would like to stress that full characterization of any real transducer technically requires measuring the transducer output using four different loads (three, if the transducer is reciprocal; see Appendix 2). Simply determining the voltage-displacement relations in unloaded cells is insufficient to draw strong conclusions regarding the speed of the OHC motor, which depends on the mechanics of the surrounding tissue. In this regard, theoretical studies well rooted in fundamental physics suggest significant enhancement of OHC high-frequency responses in situ [22, 26, 27, 36], and both in situ and in vivo recordings of electrically evoked OHC responses [41, 67, 68] reveal no problematic low-pass OHC responses, at least up to CF. Indeed, recent in vivo recordings in the gerbil base (CF $\approx$ 40 kHz) indicate that the near-CF cycle-by-cycle elongation of the OHC is $\sim$30 dB larger than the motion of the OHC basal pole where it connects to the supporting cells [69].

### OHCs as Regulators?

Those who regard the long OHC time constant as precluding prestin-based cochlear amplification attribute alternative functionality to the OHCs. For example, it is now established that the asymmetric form of the MET transduction function produces large low-frequency (quasi-DC) distortions at the OHC output [11, 15]. These quasi-DC motions track the BM-response envelope and are often comparable to or larger than the component at the stimulus frequency (i.e., the AC response). van der Heijden and colleagues have therefore proposed that the OHCs act not primarily to boost cochlear responses via fast, cycle-by-cycle forces but to slowly modulate the characteristics of cochlear wave propagation by leveraging OHC DC responses to regulate the effective material properties of the organ of Corti (e.g., its internal damping). In this view, the OHCs serve, in effect, as automatic gain controllers [9, 11] (AGCs). Notwithstanding the fact that AGC theories have been criticized and found wanting on other grounds [70], the proposal raises multiple theoretical and empirical questions, a handful of which are reviewed below.

Can the regulatory framework be made internally consistent? Although tissue motions in the “OHC region” of the organ of Corti appear low-pass relative to that of the BM [11, 15], transfer functions measured at this location are strongly peaked relative to stapes motion or ear-canal pressure (peak-to-tail ratios are $\sim$30 dB in the mouse), with AC responses in the OHC region being larger than BM motion near CF [15, 71], in good agreement with the analysis presented above. When discussing these results within the regulatory framework, van der Heijden and colleagues interpret one and the same high-frequency motions in two incompatible ways: first, as the direct result of OHC electomotility—when the immediate goal is to highlight the existence of low-pass filtering by the OHC membrane [11]—and, on the flip side, where the goal shifts to explaining cochlear sensitivity and dynamic range without recourse to cycle-by-cycle amplification, these same motions are hypothesized to result from unspecified (but non-electromotile) forces produced by “internal” waves shaped by “structural constraints” [72].

Theories that propose a central, regulatory role for the DC response of the OHC share fundamental tenets with Kolston’s “impedance-reduction” hypothesis [73]. This hypothesis posits that OHCs act to regulate the reactive and/or resistive components of the partition impedance (i.e., its stiffness and/or damping), without boosting the energy carried by the traveling wave (e.g., [9, 74]). Kolston himself ultimately determined that impedance-reduction models do not fit the data [75], concluding that the measured BM phase response (or, equivalently, cochlear traveling-wave dispersion) implies some form of energetic boost to the wave. This result is no mere model-dependent speculation: wave dispersion and energy accumulation are not independent of one another, but are coupled via the Kramers-Kronig relations imposed by causality [2].

How does the hypothesized regulation actually work? Concrete mechanisms that might couple quasi-DC OHC contractions to the traveling wave have yet to be identified, and proposed AGC models are at odds with the experimental data [70]. Wave physics implies that the traveling pressure wave couples primarily to the AC component of tissue motion, with the coupling strength increasing with frequency below CF (e.g., [76–79]). Empirically, OHC activity couples most strongly to the traveling wave in a narrowband region just below CF (in spatial terms, just basal to the peak) [55, 56, 71]. Furthermore, electrically evoked responses are broadband near the apical surface of the OHC but narrowband on the BM [80], suggesting that the coupling between OHCs and the BM is indeed weak at low frequencies. Any proposed DC-coupling mechanism would face a number of other challenges. How, for example, would it ensure that the large depolarizations (contractions) of the OHC that occur at high sound levels in vivo produce nearly the same effect on cochlear gain as the hyperpolarization (expansion) that occurs after death? And how can the frequency-dependent, non-monotonic growth of OHC DC responses, which can even change polarity across levels [9], be squared with the regular and comparatively simple level- and frequency-dependence of measured cochlear gain?

And, finally, is the regulatory mechanism robust to noise? Proponents of a purely regulatory role for OHC electromotility argue that the evolution of cochlear power amplification would be counterproductive for hearing, since the amplifier would inevitably boost the internal noise and thereby degrade the intracochlear signal-to-noise ratio [9], making signal detection more difficult. Puzzlingly, however, this argument overlooks the fact that the amplifier would also boost the signal. Indeed, according to transmission-line theory—a mathematical framework successfully used to represent cochlear wave propagation in 1, 2, and 3 dimensions [61, 77, 79, 81]—spatially distributed (cascaded) wave amplification boosts the input signal, which is spatially coherent, more than the internal noise, which is not [82, 83]. Passive transmission lines, in which the spatial gradient of wave power is negative, manifest the opposite behavior: input signals are attenuated more than the internal noise. Since internal noise increases dramatically at low frequencies—see Fig. 2a, but also consider that additional noise sources with pink (1/*f*) or brown ($1/f^2$) spectra are omnipresent [84]—regulatory mechanisms that rely on quasi-DC responses appear problematic.

### Real-Life Examples Illustrate the Problem with the *RC* Problem

The existence of spontaneous otoacoustic emissions [85, 86], the presence of prestin-dependent active motion and amplification in vivo, and the physical reasoning embodied by modern cochlear models (e.g., [87]) all indicate that OHCs generate significant power at the signal frequency. Nevertheless, the role of cycle-by-cycle OHC electromotility remains hotly debated. As highlighted here, the arguments questioning the role of OHC motility generally sidestep the principal issue—whether OHC motions are large enough to have significant functional effects—to focus instead on two perhaps overhasty conclusions: (i) that prestin-based cochlear power amplification must be inconsequential when the stimulus period is longer than the time constants characterizing prestin kinetics and the OHC membrane; and (ii) that because distortions and low-pass filtering combine to produce OHC DC responses that are larger than the AC response, these DC responses must, ipso facto, be of paramount importance for cochlear function.

**Fig. 4:**
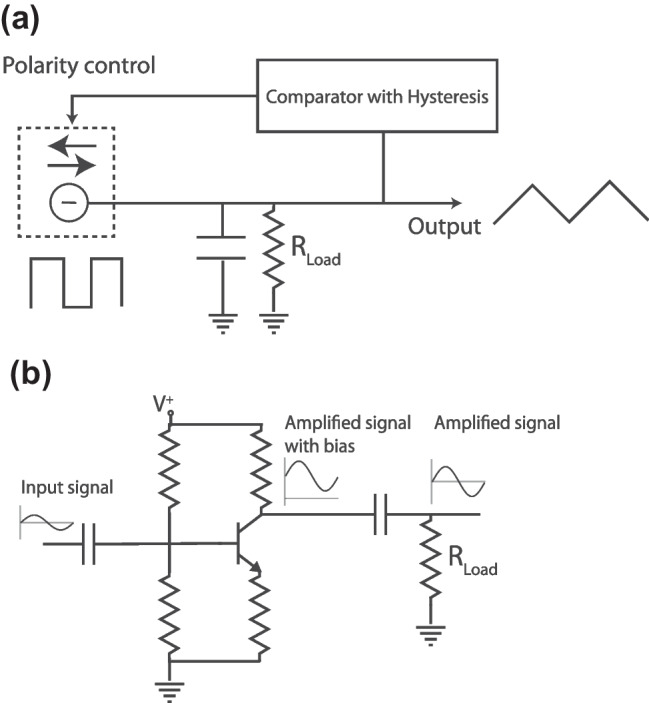
**a** Schematic of a current-controlled oscillator, whose operational frequency range is not limited by the time constant of the charging element (in this case a real capacitor, whose *RC* time constant is very large), but by the available current and the required voltage swing at the output. **b** Schematic of a textbook signal amplifier. The signal at the output terminal (collector) of the transistor consists of a DC bias plus an amplified version of the input signal. The series capacitor at the output terminal, together with the load resistance $R_\mathrm {Load}$, acts as a high-pass filter, removing the DC bias from the amplified signal (AC coupling). Although the DC response at the transistor output can be larger than the AC component, the AC coupling ensures that the DC response has no functional consequences when the amplifier is embedded in a larger system. Notwithstanding the obvious differences between these example circuits and cochlear OHCs (e.g., OHCs contain no auxiliary circuitry that switches the current polarity depending on voltage, as in the electronic oscillator; and cochlear wave amplification requires the cooperation of multiple OHCs, whereas signals can be greatly amplified by only a single transistor), the examples nevertheless provide real-life analogies that help elucidate mechanisms underlying OHC function

Figure 4 presents two popular electronic circuits whose operation illustrates why these arguments are hardly dispositive. The operation of the current-controlled (triangle) oscillator (Fig. 4a) is straightforward. A constant current charges a capacitor. When the voltage reaches a desired maximum value, an auxiliary circuit inverts the polarity of the current, which steadily discharges the capacitor. When the capacitor voltage reaches a desired minimum value, the current polarity is again reversed, recharging the capacitor once more. And so on, ad infinitum. The voltage output of the circuit is a triangular waveform, whose frequency depends on the current source, the capacitance, and the desired voltage swing. Although in an ideal oscillator the load resistance ($R_\mathrm {Load}$) and therefore the *RC* time constant of the quiescent oscillator are infinite—in practice both are limited by parasitic conductances and other non-ideal circuit elements—the circuit can oscillate at very high frequencies. Thus, the operational frequency range is limited not by the *RC* time constant but by the available current and the required voltage swing at the output. We suggest that the same is true of the OHC in vivo.

The second circuit is a textbook, single-transistor amplifier (Fig.  4b). The signal at the amplifier output (transistor collector) consists of the amplified input (transistor base) plus a DC offset that is typically larger than the amplified signal. The introduction of a DC bias in the signal path is often undesirable (e.g., in audio applications). In addition, the amplifier can introduce significant low-frequency noise. These problems are commonly solved by connecting a capacitor in series with the circuit output—the capacitor acts as a high-pass filter (whose corner frequency is determined by the capacitance and the finite input impedance of the following stage, represented in Fig.  4b by the load resistor $R_\mathrm {Load}$) that removes the DC bias and attenuates low-frequency noise (AC coupling). Just as with the OHC, the DC response of the amplifier can be larger than the AC response. However, AC coupling ensures that the DC response remains squelched and functionally unimportant. The evidence to date suggests that OHC motions are AC coupled to the traveling wave on the BM.

Arguments suggesting that the low-pass filtering action of the OHC membrane precludes effective cycle-by-cycle wave amplification in the cochlea evidently fall prey to the fallacy of composition—mistaking the behavior of the parts (OHCs or their subcomponents, such as prestin) for that of the whole (the spatially distributed cochlear amplifier). For example, one can easily obtain high-, band-, and low-pass transfer functions by cascading multiple low-pass filters and mixing their responses. In a similar way, the cochlear amplifier can be thought of as emerging from the “mixed” responses of multiple OHCs. Mixing occurs hydrodynamically, via the fluids and other coupling mechanisms, and each coupling mechanism has its own frequency (or wavelength) dependence. Perhaps it should not be surprising that the frequency dependence of cochlear amplification differs from that of the isolated OHC.

In this regard, previous modeling efforts [53, 88] reveal that the OHC-generated active pressure necessary to fit the data in simple 3D physics-based cochlear models is low-pass filtered relative to the motion of the BM. The OHC action in traveling-wave models of cochlear amplification therefore appears different from the sharply tuned force production posited by the oscillator models often employed to deduce the relations between OHC biophysics and cochlear amplification (e.g., [36]). Just as the response of a loudspeaker depends not only on the speaker itself, but also on the cabinet in which the speaker is mounted and on the room where the loudspeaker is placed and assessed, so any satisfactory understanding of cochlear amplification requires simultaneous consideration of the biophysical properties of the OHC (the speaker), the organ of Corti that embeds and supports it (the cabinet), and the tapered, fluid-filled cochlear duct (the room) wherein it and a thousand others gather to sing.

## Figures and Tables

**Fig. 5:**
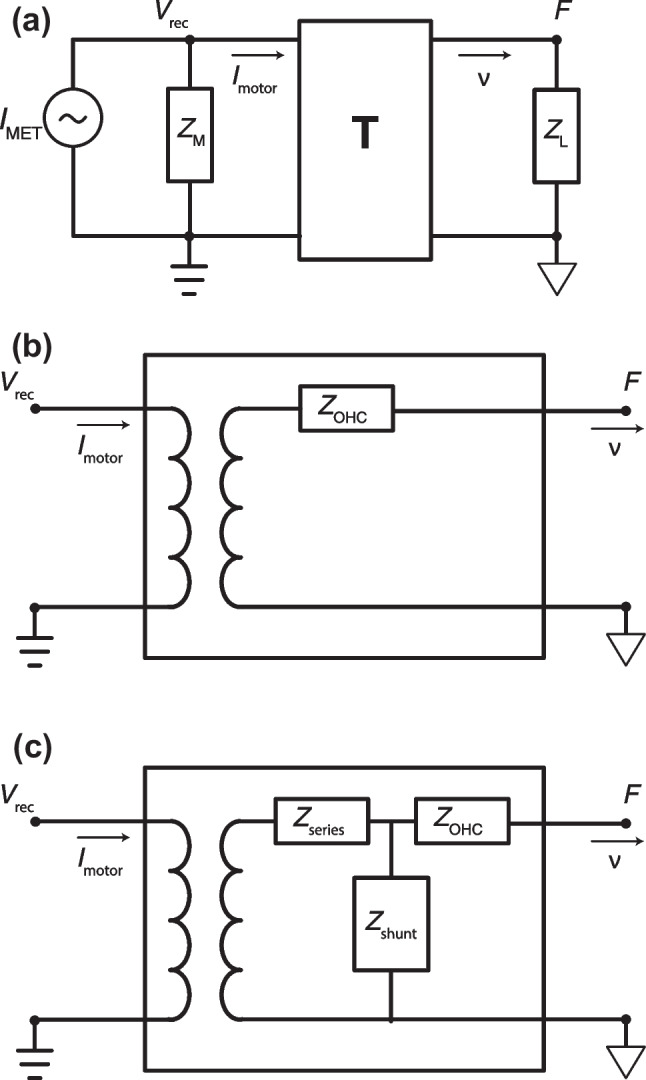
**a** General linearized model of OHC electromotility, where the electrical-to-mechanical process is represented by an unspecified two-port network. **b**,** c** Electrical representations of the two contrasting interpretative models of OHC reverse transduction that underlie the current debate on the speed of of the OHC motor. **b** According to one interpretation, the frequency-dependencies observed in OHC responses in vitro are meaningfully explained by the cascade of a nearly ideal piezo-electric transformer with the intrinsic OHC load (i.e., OHC stiffness, viscosity, and nonlinear capacitance; see [23, 26, 27]). **c** In the second interpretation, the observed frequency-dependencies mainly reflect the kinetics of the OHC prestin motor, which cannot be treated as a frequency-independent nearly ideal piezoelectric transformer [10, 40, 91]. Using consolidated electromechanical analogies, frequency-dependency and non-ideal behavior of the OHC-prestin motor can be mathematically represented by introducing frequency-dependent shunt and series impedances in the two-port network representing electrical-to-mechanical transduction (here, for simplicity, only one series and shunt impedances are drawn)

## Appendix 1. (Over)linearization of the OHC membrane

Mechanically gated mechanoelectrical transduction (MET) channels drive the electrical response of the OHC basolateral membrane. The electrical impedance of the basolateral membrane (Fig. 1a) is determined primarily by (i) outward K$^+$ currents, whose action is collectively represented by a voltage-dependent resistor [13]; (ii) the membrane capacitance; and (iii) a complex impedance that captures the electrical effects of the mechanical load on the OHC [22]. Motion of the OHC stereocilia modulates the ionic current that flows through the MET channels:

6 $$\begin{aligned} I_\mathrm {MET}=g(\mu )(V_\mathrm {M}-V_\mathrm {EP})\;, \end{aligned}$$

where $\mu$ is the stereocilia displacement, $g(\mu )$ is the transduction function (units of ${[\Omega ]^{-1}}$), $V_\mathrm {EP}$ the endocochlear potential, and $V_\mathrm {M}$ the OHC membrane potential. For simplicity, we ignore the still unresolved effects of adaptation [89] on $\mu$ and *g*. Accounting for such effects in a linearized model amounts to high-pass filtering the model input and does not modify the conclusions presented in the main text.

Rewriting $V_\mathrm {M}$ as the sum of the resting ($V_\mathrm {rest}$) and receptor ($V_\mathrm {rec}$) potentials yields

7 $$\begin{aligned} I_\mathrm {MET}=g(\mu )(V_\mathrm {rest}+V_\mathrm {rec}-V_\mathrm {EP})\;. \end{aligned}$$

In the small-signal limit ($|V_\mathrm {rec}|\ll |V_\mathrm {rest}-V_\mathrm {EP}|$), the dependence of $I_\mathrm {MET}$ on $V_\mathrm {rec}$ can be neglected, and $I_\mathrm {MET}$ becomes proportional to the transduction function, $g(\mu )$. When the stereocilia displacement $\mu$ is a sinusoid of amplitude *A* and frequency *f*, Taylor expansion of $g(\mu )$ leads to

8 $$\begin{aligned} \begin{aligned} I_\mathrm {MET}\,\approx \;&I_\mathrm {MET}(0) + \bar{I}_\mathrm {MET}(A) + \tilde{I}_\mathrm {MET}(A)\sin (2\pi f t)\; + \\&\qquad + O(A^2\sin (4\pi f t))\;, \end{aligned} \end{aligned}$$

where $\bar{I}_\mathrm {MET}$ and $\tilde{I}_\mathrm {MET}$ represent, respectively, the DC and AC components of the MET current; the *A*-dependent DC component arises from the asymmetry of the transduction function around $\mu =0$.

In line with studies arguing against the relevance of high-frequency OHC electromotility, we over-simplify the model of the OHC membrane to obtain the *RC* circuit presented in Fig. 1b. This “straightforward linearization” (cf. [11]) ignores leading-order linear terms that emerge when properly linearizing basolateral currents (see [90]), and it is therefore not a true linearization. In addition, this model ignores the contribution of the mechanical load ($Z'_\mathrm {L}$ in Fig. 1a) to the electrical properties of the OHC, which is likely significant (e.g., [22, 26, 27]). Nevertheless, the over-simplifications provide useful insight. In particular, the electrical impedance of the OHC membrane is simply

9 $$\begin{aligned} Z(f)=\frac{R}{1+i 2 \pi f RC}\;, \end{aligned}$$

whose associated 3-dB cut-off frequency is $f_c=[2\pi {RC}]^{-1}$, with *R* representing the resting basolateral resistance. Note that in previous literature, the resting MET conductance is sometimes included in the calculation of *R* (e.g., [13]). The definition of the *RC* time constant requires a linearized model, and linearization when the input of the model is the vibration of the sterocilia leads one to represent the MET channels as a current generator (Fig. 1b). We therefore find it more informative to exclude the MET resting conductance from the calculation of *R*. Indeed, while the MET resting current indirectly contributes to the filtering property of the OHC membrane (by determining resting potential and hence the resting basolateral conductance), once the parameters are“fixed” in the model, the oscillating (AC) component of the OHC potential is independent of the MET resting current.

## Appendix 2. The OHC-Motor Speed Problem, Simplified

In the small-oscillation limit, where approximate linearity of the motor can be assumed, transduction from one domain to another can be characterized using a two-port network. For the case of OHC electrical-to-mechanical transduction, the network relates four quantities: the receptor potential, $V_\mathrm {rec}$; the current absorbed by the prestin-motor, $I_\mathrm {transduction}$; the OHC mechanical force, *F*; and the elongation velocity, $\nu$. Figure 5a shows a general circuit model of OHC mechanical transduction. The electrical and mechanical domains appear on on the left and right sides of $\mathbf{{T}}$, respectively. The electrical domain includes the MET current generator and the membrane impedance, $Z_\mathrm {M}$. The mechanical domain includes the impedance representing the mechanical load on the OHC, $Z_\mathrm {L}=F/\nu$; effects of stiffness and viscosity of the OHC membrane (i.e., “self-load”) are conveniently incorporated in the two-port network (see below). The relations between the voltage, current, force, and velocity can be expressed in various equivalent manners in the frequency domain. Here we find it convenient to employ the *ABCD* matrix:

10 $$\begin{aligned} \begin{bmatrix} V_\mathrm {rec}\\ I_\mathrm {transduction}\end{bmatrix} =\mathbf{T}\begin{bmatrix}F \\ \nu \end{bmatrix}, \end{aligned}$$

with

11 $$\begin{aligned} \mathbf{T}= \begin{bmatrix} A &{} B \\ C &{} D \end{bmatrix}\;. \end{aligned}$$

The frequency-dependence of the complex-valued parameters *A*, *B*, *C*, and *D* is left implicit for simplicity. Note that this general model relies on no assumption (e.g., on the efficiency of the piezo-electric motor) but linearization. The *ABCD* coefficients describe the following relations

12 $$\begin{aligned} \begin{matrix} \displaystyle A=\left. \frac{V_\mathrm {rec}}{F}\right| _{\nu =0} &{} \displaystyle B=\left. \frac{V_\mathrm {rec}}{\nu }\right| _{F=0} \\ \displaystyle C=\left. \frac{I_\mathrm {transduction}}{F}\right| _{\nu =0} &{} \displaystyle D=\left. \frac{I_\mathrm {transduction}}{\nu }\right| _{F=0} \end{matrix}\;, \end{aligned}$$

where the frequency dependencies introduced by the prestin motor and OHC self-load are encapsulated in the *ABCD* parameters.

This treatment reveals the dynamics of the OHC motor can in principle be characterized by estimating voltage, current, velocity, and force when mechanical blocking the cell ($\nu =0$) or with zero external load ($F=0$). Unfortunately, this is easier said than done. For example, it would be difficult to monitor how $I_\mathrm {transduction}$—which represents the fraction of MET current that does not flow through the basolateral membrane—varies depending on the mechanical load given the presence of basolateral currents that depend nonlinearly on voltage and load [32]. Additionally, it is not easy to ensure that $F\approx 0$, given that OHC motion must be measured while the cells are immersed in physiological solution and clamped on one end. Furthermore, $\mathbf{T}$ depends on a myriad of experimental parameters (see, e.g., [10, 16, 24]). As a result of these complexities, only the matrix element *A* has been measured to date [35], and even that only for a particular combination of experimental parameters whose physiological relevance has been questioned [10]. Note, though, that at each frequency characterizing $\mathbf{T}$ boils down to determining four complex-valued parameters, technically requiring four independent measurements (three, if the system is known to be reciprocal, so that $\det \mathbf{T}=1$).

From the perspective of the two-port model, the current debate about the speed of the OHC motor can be encapsulated by the two different interpretative models of $\mathbf{T}$ illustrated in Fig. 5b, c. The first interpretation (Fig. 5b), more favorable to high-frequency OHC operation, posits that the OHC transduction machinery operates similarly to an ideal piezo-electric transformer. In this view, the frequency-dependencies observed in the experimental data (e.g., the frequency-dependent voltage elongation or the kinetics of charge movement deduced from recordings of OHC nonlinear capacitance [92, 93]) depend heavily on the intrinsic and applied loads [27]. Thus, the load impedance plays a primary role in setting the both the high-frequency limit of OHC electromotility (see also [23, 26]) and the frequency range where the OHC delivers mechanical power to the sensory tissue [31, 94]. In addition, it has been suggested that interaction with the load might create a piezo-electric resonance that effectively transforms the low-pass *RC* membrane into a resonant band-pass filter (e.g., [22, 36]).

The second interpretation supposes, in effect, that the frequency dependence of electromotility depends not so much on the cellular load as on speed limits intrinsic to the OHC motor, such as those potentially arising from constraints on charge motion in the basolateral membrane [10, 40] and/or the finite reaction time of the prestin protein [91, 95]. In this view, OHC performance is not easily modified or controlled by interactions with the mechanical environment; the motor itself is the main cause of the observed behavior. Physically, these limiting factors can be represented by adding series ($Z_\mathrm {series}$) and shunt ($Z_\mathrm {shunt}$) impedances on the right-hand side of an ideal transformer (Fig. 5c). The effect of these impedances is to decrease the force and velocity of the OHC in a frequency dependent manner. Studies supporting this interpretation typically adopt different but mathematically equivalent models, such as voltage-dependent, two-state models of prestin conformation (e.g., [24]). (Note that such additional impedances can equivalently be drawn on the left-hand side of the transformer, and, indeed, the non-ideal behavior of real transformers is conventionally referred to the left side. Here, however, we draw them on the right-hand side in order to highlight their origin in the kinetics of the prestin motor.) As an example, including a series inductance or a shunt capacitance on the right side of the transformer would effectively introduce a low-pass filter in the relationship between membrane voltage and OHC elongation velocity.

In either interpretation of OHC electromechanical transduction—or any that might lie “in-between”—the effects of the transduction machinery on the membrane impedance are conveniently encapsulated by an equivalent impedance $Z'_\mathrm {L}$, as in Fig. 1a. Indeed, from Eqs. (10 and 11), we have that

13 $$\begin{aligned} Z'_\mathrm {L}=\frac{AZ_\mathrm {L}+B}{CZ_\mathrm {L}+D}\;. \end{aligned}$$

Accounting for the load, the effective membrane impedance $Z_\mathrm {eff}$ becomes

14 $$\begin{aligned} Z_\mathrm {eff}=\frac{Z_\mathrm {M}Z'_\mathrm {L}}{Z_\mathrm {M}+Z'_\mathrm {L}}\;. \end{aligned}$$

For an *RC* membrane with corner frequency $f_c=[2\pi RC]^{-1}$, the effective impedance becomes

15 $$\begin{aligned} Z_\mathrm {eff}=\frac{RZ'_\mathrm {L}}{R+Z'_\mathrm {L}(1+if/f_c)}\;. \end{aligned}$$

From this equation, one can readily appreciate that when $f\gg f_c$, the *RC* filtering acts to decrease the effective impedance by 6 dB/octave. In other words, at high enough frequencies, the membrane shunt capacitance always reduces the OHC membrane potential by 6 dB/octave relative to a hypothetical OHC model where there the shunt capacitance is somehow removed.

## Appendix 3. Estimate of Inherent OHC Electric Noise

Following [9], we consider the inherent electric noise at the OHC output as primarily determined by thermal noise and by shot noise caused by the random gating of MET channels. The analysis we present here differs from theirs in that we account for the frequency-dependence of OHC noise and provide a more detailed treatment of the shot-noise case. OHC noise is conveniently quantified by modeling noise sources as current generators driving the OHC membrane (Fig. 1b). The equivalent thermal noise current source has power spectral density

16 $$\begin{aligned} S_{I,\mathrm {TN}}={4{k_b T/R}} \qquad [{\mathrm{A}^2/\mathrm{Hz}}]\;, \end{aligned}$$

where $k_b$ is the Boltzmann constant, *T* the absolute temperature, and *R* the OHC resistance. The thermal-noise spectral density manifest in OHC voltage is simply

17 $$\begin{aligned} S_{V,\mathrm {TN}}(f)=S_{I,\mathrm {TN}}|Z(f)|^2 \qquad [{\mathrm{V}^2/\mathrm{Hz}}]\;, \end{aligned}$$

which describes a random voltage with RMS amplitude $\sqrt{k_bT/C}$ and noise bandwidth 1/4*RC*.

The second source of inherent OHC electrical noise is the shot noise associated with the random opening and closing of the MET channels. We assume that the number of open channels at any instant is described by a binomial distribution [96] and that, for simplicity, the opening and closing of the MET channels exhibit first-order dynamics with activation time constant $\theta$. In this case, the autocorrelation of the stochastic process describing the current flowing through the MET channels is (see e.g., [84])

18 $$\begin{aligned} c(t)=\frac{1}{2}{Np(1-p)i^2} e^{-|t|/\theta }\;, \end{aligned}$$

where $i=I_\mathrm {MET}/N$ is the current mediated by a single MET channel, *N* the number of MET channels, and *p* the channel’s open probability. The current power spectral density is the Fourier transform of the autocorrelation function (Eq. (18)):

19 $$\begin{aligned} S_{I,\mathrm {SN}}(f)= Np(1-p)i^2\frac{\theta }{1+(2\pi \theta f)^2}\;. \end{aligned}$$

The voltage spectral density is then found simply by solving the circuit in Fig. 1b:

20 $$\begin{aligned} S_{V,\mathrm {SN}}=S_{I,\mathrm {TN}}(f)|Z(f)|^2. \end{aligned}$$

Although the resting value of *p* remains controversial—in vitro estimates range from roughly 10 to 50% [13, 14]—relatively strong rectification and even-order harmonics are observed in mechanical responses from the OHC region in both the base and apex of the mouse [15, 97], and in the base of the gerbil as well [11], suggesting that *p* is relatively small (i.e., 20–30%). For our calculations, we therefore assume $p=30$%, based on the recordings of [15], and $N=100$ (i.e., $\sim$60 tip-links with on average of 1.65 channels per tip-link [33]).

## Appendix 4. Membrane Low-Pass Filtering Reduces Noise from MET Channel Gating

To consider the effect of membrane low-pass filtering on the shot noise caused by the stochastic gating of the MET channels, it proves convenient to reason in terms of noise bandwidths. In particular, a white noise source subject to first-order low-pass filtering (such as the shot noise current, see above) is commonly characterized using the low-frequency asymptotic value of its spectral density $\bar{S}=\lim S(f)_{f\rightarrow 0}$ and its noise bandwidth $\Delta f=1/4\tau$, where $\tau$ denotes the low-pass filter time constant. Conveniently, the total noise power (as computed using the complete set of equations) equals the product of the asymptotic spectral density and noise bandwidth ($\bar{S}\Delta f$).

The MET activation time constant $\theta$ is much smaller than the membrane *RC* time constant. Indeed, the MET channel activation time constant is so small that experimental limitations have so far largely precluded its measurement [98]. Therefore, the decline of noise power at high frequencies—the term $1/[1+(2\pi \theta f)^2]$ in Eq. (19)—plays a secondary role for determining the overall power of the OHC shot-noise voltage (as this occurs at frequencies where the membrane low-pass filter significantly attenuates the voltage power). When calculating power, shot-noise voltage can thus be approximated as a first-order low-pass filtered noise with an asymptotic spectral density

21 $$\begin{aligned} \bar{S}_{V,\mathrm {SN}}\approx {S}_{I,SN}(0)|Z(0)|^2,\; \end{aligned}$$

and noise bandwidth 1/4*RC*. The shot-noise voltage power can then be calculated as the product of the asymptotic spectral density (Eq.  (21)) with noise bandwidth:

22 $$\begin{aligned} {P}_{V,SN}\approx {S}_{I,SN}(0)|Z(0)|^2\frac{1}{4RC}\;. \end{aligned}$$

The previous equation can be written as

23 $$\begin{aligned} {P}_{V,SN}\approx Np(1-p)i^2R^2\frac{\theta }{4RC} = Np(1-p)(\frac{V_\mathrm {max}}{2N})^2\frac{\theta }{RC}\;, \end{aligned}$$

where $V_\mathrm {max}=RNi$ indicates the theoretical maximum receptor potential, when all MET channels are held open.

Equation (23) demonstrates that the shot noise at the OHC output is determined not only by “quantization” ($V_\mathrm {max}/N$), but also by the ratio between the MET-activation and *RC* time constants (${\theta }/{RC}$). In particular, were *RC* short, comparable to $\theta$, the shot-noise voltage power would be approximately proportional to the power of the shot-noise current. As it turns out, however, $RC\gg \theta$, significantly reducing the voltage shot-noise power. By taking $\theta =4\,$ $\mu$s [99] and $RC=50\,$ $\mu$s, corresponding to a corner frequency of $\sim$3 kHz, one finds that membrane low-pass filtering reduces the shot-noise power by a factor of approximately 12.5 (11 dB) at the OHC output.
